# P-412. Management of Delayed Sternal Closure and Infection Prevention: A National Survey

**DOI:** 10.1093/ofid/ofaf695.629

**Published:** 2026-01-11

**Authors:** Madhuri Kashyap, Ishminder Kaur, Myke Federman

**Affiliations:** University of California, Los Angeles, Los Angeles, CA; Mattel Children's Hospital at UCLA, Los Angeles, CA; UCLA, Los Angeles, California

## Abstract

**Background:**

Delayed sternal closure (DSC) is commonly used in neonates and infants after cardiac surgery to support hemodynamic and respiratory stabilization. Over 10% of pediatric cardiac surgical patients undergo DSC. Sternal wound infections occur in 3.5%–18% of cases and are associated with increased mortality, prolonged hospitalization, and costs. Despite the clinical impact, antimicrobial prophylaxis practices vary widely, with no standardized guidelines on agent selection, duration, or adjunctive strategies.

**Methods:**

Pediatric Infectious Diseases and Cardiac ICU teams at UCLA developed a 20-item REDCap survey to assess institutional DSC infection prevention practices. Questions covered antimicrobial and antifungal use, timing and duration of prophylaxis, skin and nasal decolonization (e.g., CHG, mupirocin), and adjunctive strategies (e.g., visitor restrictions, wound coverage, hygiene practices). The survey was emailed to 70 pediatric cardiac ICU medical directors. Email addresses were collected to track institutional roles. No patient-level data were obtained. The study was IRB-exempt, with a $50 raffle incentive for two respondents. Biweekly reminders were sent over two months. The survey required ∼10 minutes to complete.

**Results:**

Eighteen institutions responded, reporting an average of 286 cardiopulmonary bypass cases per year. In 72.2%, the sternum remained open for 48–72 hours postoperatively. Preoperative CHG baths were used by 83.3% of centers; nasal mupirocin was commonly employed. Cefazolin was the primary prophylactic agent, although broader-spectrum antibiotics were frequently added for DSC. Antibiotics were continued for the duration of the open chest in 83% of cases. Antifungal prophylaxis was rare, used only in select high-risk scenarios. Adjunctive measures—such as Ioban use (67%), patient-specific stethoscopes, and sterile probe covers—varied. Most institutions (60%) reported infection rates in line with national benchmarks, while 40% reported lower-than-average rates.

**Conclusion:**

Wide variability in infection prevention practices following DSC underscores the need for standardized, evidence-based protocols to reduce unnecessary antimicrobial use and improve outcomes.

**Disclosures:**

All Authors: No reported disclosures

